# A rare variant at 11p13 is associated with tuberculosis susceptibility in the Han Chinese population

**DOI:** 10.1038/srep24016

**Published:** 2016-04-01

**Authors:** Cheng Chen, Qi Zhao, Yi Hu, Yan Shao, Guoli Li, Limei Zhu, Wei Lu, Biao Xu

**Affiliations:** 1 School of Public Health, Fudan University, Shanghai, China; 2 Department of Chronic Communicable Disease, Center for Disease Control and Prevention of Jiangsu Province, Nanjing, China; 3 Key Laboratory of Public Health Safety (Fudan University), Ministry of Education, Shanghai, China; 4 Department of Public Health Sciences (Global Health / IHCAR), Karolinska Institutet, Stockholm, Sweden

## Abstract

Genome-wide association studies (GWASs) have yet to be conducted for tuberculosis (TB) susceptibility in China. Two previously identified single nucleotide polymorphisms (SNPs) from tuberculosis GWASs, rs2057178 and rs4331426, were evaluated for TB predisposition. The associations between SNPs and gene expression levels were analyzed using the genomic data and corresponding whole-genome expression of the Han Chinese in Beijing, China. Genotyping was successfully completed for 763 pulmonary TB patients and 763 healthy controls. The T allele of the rare variant rs2057178 was significantly associated with TB predisposition (χ^2^ = 14.07, *P* = 0.0002). Meanwhile, the CT genotype of rs2057178 was associated with a decreased risk of TB (adjusted OR = 0.52, 95% CI, 0.34–0.78). The CT genotype of rs2057178 was also associated with decreased expression levels of infection-related gene, suppressor of cytokine signaling 2 (SOCS2), and increased expression levels of v-maf avian musculoaponeurotic fibrosarcoma oncogene homolog B (MAFB). No gene expression levels were found to be associated with the genotype of rs4331426. We found that the rare variant rs2057178 was significantly associated with TB in the Han Chinese population. Moreover, the expression levels of MAFB and SOCS2 correlated with rs2057178 and might be potential candidates for assessing TB susceptibility.

According to the WHO 2014 global tuberculosis (TB) report^1^, China has the second largest number of incident TB cases, which accounts for 11% of the global burden. Moreover, the recent TB infection survey conducted in China indicated that one-fifth of the population had been latently infected by *Mycobacterium*. tuberculosis (*M*. tb)^2^. Although a majority of the *M*. tb-infected population will not progress to clinical TB because of host resistance to the pathogen, it was estimated that approximately 10% of infected individuals will develop TB disease during their lifetime^3^. The large reservoir of infected people is a crucial threat to China’s TB control program for achieving the END TB goal in 2035^4^. Whether a person who is latently infected with *M*. tb would become a clinical case may largely depend on the host defense against *M*. tb. The vast difference between *M*. tb infection rate and population TB incidence suggests that the human host plays an important role in preventing TB development after infection. Evidence from studies of twins also support the assumption that host genetics contribute to the susceptibility of TB^5^.

In recent years, TB candidate genes, such as the vitamin D receptor gene^6^, cytokine genes^7^, and toll-like receptors genes^8^, have been studied to explain host genetic susceptibility to TB in various ethnic backgrounds. However, due to limited sample sizes and/or the diversity of ethnicity, evidence of the association between hot spot loci of candidate genes and TB susceptibility is still rare. Genome-wide association studies (GWASs) have provided a large amount of information regarding the genetic contribution of hundreds of thousands of genomic variants to TB susceptibility^9^,^10^,^11^, and some of the variants were highly associated with TB susceptibility. However, no GWAS of TB predisposition has been conducted in China yet.

Common variants of the genome usually have little effect on common disease risk, usually conferring small odds ratios on disease risk effect. Recently, Rivas *et al.* proposed that rare variants would have a larger effect and higher penetrance in disease occurrence^12^. In this study, we selected SNPs with allele frequencies of less than 5% in the Han Chinese population and that were found to be significantly associated with TB by previous GWASs. Thus, the single nucleotide polymorphism (SNP) rs2057178 at 11p13 and the SNP rs4331426 at 18q11.2, which were identified in previous GWASs, were selected to verify their association with TB predisposition^10^,^11^. More importantly, the suggestion of Wilkinson^13^ was adopted to test the latent TB infection status of the control group to control for the exposure factor of *M*. tb infection in this study. Meanwhile, the Gene Expression Omnibus Database was used to explore the relationship between the selected SNPs and whole genome mRNA expression levels^14^,^15^. Lymphoblastoid cell lines, which carry the complete set of germline genetic material, have been instrumental, in general, as a source of biomolecules and as a system to carry out various immunological and epidemiological studies^16^. Dissemination of *M*. tb in infected persons may be connected with the initiation of adaptive immune responses, which are under strict host genetic control^17^. The transcriptome level among the lymphoblastoid cell lines may be closely associated with disease-associated genetic variants. Thus, we consider that specific gene expression levels in lymphoblastoid cell lines may be closely regulated by host genomic variants. RNAs extracted from lymphoblastoid cell lines of the 45 unrelated CHB of the HapMap Project were quantified to explore the relationship between selected SNPs and the expression levels of potential TB susceptibility genes.

## Methods

### Study subjects

This case-control study was carried out in two designated hospitals with TB control programs in Jiangsu Province, eastern China. One was the TB control center of Danyang County, and the other was the Nanjing Chest Hospital in the province’s capital city. Incident TB cases of Han ethnicity registered in these two hospitals from July 1st, 2013 to December 31st, 2014 were recruited for inclusion as cases in this study. All enrolled cases were bacteriologically confirmed by Lowenstein-Jensen (LJ) culture, and *M.* tb was identified using the p-nitrobenzoic acid (PNB) method. Meanwhile, the healthy controls were recruited from two communities from Danyang County during the same study period. All control candidates underwent X-ray examination. Sputum culture was provided if the potential controls reported having TB-like clinical symptoms. Only subjects with normal X-ray manifestation, negative LJ culture, if tested, and no comorbidity with other infectious diseases (such as HIV/AIDS and hepatitis B virus) were eligible as healthy controls. All of the controls were of Han ethnicity, and they were 1:1 matched to the cases by age (±5 years) and gender. In total, 764 TB cases and 764 healthy controls were recruited for the study. All experimental protocols in this study were approved by the Institutional Review Board of the Center for Disease Control and Prevention of Jiangsu Province, and written informed consent was obtained from each participant before the study. Additionally, all of the methods in this study were carried out in accordance with the approved guidelines.

### Interferon-Gamma Release Assay

The interferon-gamma release assay (QuantiFERON-TB Gold In-Tube [QFT; Qiagen, Valencia, CA, USA]) was used to test the latent TB infection (LTBI) status of the controls. QFT was performed according to the instructions provided by Qiagen^18^.

### Genotyping of rs2057178 and rs4331426

The restriction fragment length polymorphism (RFLP) method was used for the genotyping. The sequences of the primers used to amplify the PCR fragment of rs2057178 were as follows: 5′-TCC ATT GGC CTG AAC TGG AT-3′ (forward); 5′-TGG CCT CCA GTT CTT TAG CA-3′ (reverse). A 186 base pair PCR fragment was amplified by the primers. The restriction endonuclease enzyme *StuI* (New England BioLabs, inc., Ipswich, MA, USA) was used to digest the PCR fragment. The presence of the C allele results in two fragments: one fragment of 125 base pairs in length and one fragment of 61 base pairs in length. The presence of the T allele results in a single fragment of 186 base pairs in length. The PCR amplification fragment for rs4331426 was 250 base pairs in length, and the sequences of the amplification primers were as follows: 5′-AAG GGT GTT GTT CTG TTT CTA GA-3′ (forward), 5′-TGT TGC ACC ACC TCT TGT AGA-3′ (reverse). The restriction endonuclease enzyme *HhaI* (New England BioLabs, inc., Ipswich, MA, USA) was used to digest the PCR fragment. The presence of the G allele results in two fragments: one fragment of 202 base pairs in length and one fragment of 48 base pairs in length. The presence of the A allele results in a single fragment of 250 base pairs in length.

### Genotypic data of rs2057178 and rs4331426 of the 45 Han Chinese in Beijing (CHB) from the HapMap Project and whole-genome expression levels from the Gene Expression Omnibus of PubMed

The genotypic data of rs2057178 and rs4331426 were extracted from the HapMap Genome Browser Release #28 (phases 1, 2 & 3-merged genotypes and frequencies), and the genotypes of each SNP for the 139 CHB individuals were derived from this database. The DNA samples were prepared from blood samples collected from individuals living in the residential community at Beijing Normal University. All of the samples are from unrelated individuals who identified themselves as having at least three out of four Han Chinese grandparents.

Finally, 45 CHB provided validated genotypic data of the two SNPs. Using mRNAs extracted from lymphoblastoid cell lines for the corresponding 45 CHB, the Gene Expression Omnibus (GEO) of PubMed (accession number GSE6536) was used to analyze the relationship between the two SNPs and the whole-genome mRNA expression levels of the 47,293 genes^14^,^15^.

### Statistics

An unpaired Student *t* test was applied to numerical variables, whereas the differences in categorical variables were tested using the χ^2^ test. The Cochran-Armitage trend test was used to compare the genotype dosage among the TB cases and controls. Hardy-Weinberg equilibrium (HWE) was assessed by Pearson χ^2^ test. The strength of associations between genotypes and TB were estimated by odds ratio (OR) and its 95% confidence interval (95% CI) through univariate and multivariate logistic regression analyses adjusted for age and gender. A *P* value of less than 0.05 was considered statistically significant. The relationships between the genotypes of the SNPs and the gene expression levels of the 45 CHB were analyzed by the online software GEO2R based on the moderate *t* test. Meanwhile, the usual *t* test was also applied to analyze the relationship between the genotypes and the gene expressions. The Benjamini & Hochberg (False discovery rate) was adopted for the multiple comparison correction^19^. The significance level for gene expression among different genotype groups was *P* < 0.20^20^. The value of fold change (FC) was calculated for showing the fluctuation of the gene expression among different genotype groups. The software Quanto (Version: 1.2.3, South California, USA) was employed to calculate the power of the OR on TB risk. All of the analyses were performed using Stata software (Version 13.0, StataCorp, Texas, USA).

## Results

A total of 763 TB cases and 763 controls were included in this analysis, with one case and one control failing the genotyping. The mean ages for TB cases and controls were 49.17 ± 17.48 years and 52.03 ± 17.33 years, respectively. The QFT results showed that the positive rate of LTBI for the controls was 23.1% (176/763). As shown in Table 1, the minor allele (T allele) frequency of rs2057178 was 0.048 in the TB cases and 0.027 in the healthy controls (χ^2^ = 14.07, *P* = 0.0002). As the minor allele of rs2057178 was less than 0.05 and the TT genotype was only found in six subjects, the TT genotype among TB cases and controls showed a decreased risk of TB without reaching significance (adjusted OR = 0.56, 95% CI, 0.10–3.10). However, the CT genotype was significantly associated with a decreased risk of TB (adjusted OR = 0.52, 95% CI, 0.34–0.78). The dominant model (CT + TT vs. CC) demonstrated a protective effect on TB (adjusted OR = 0.52, 95% CI, 0.35–0.78). Based on the T allele frequency (0.048) of rs2057178 and the estimated TB prevalence (51/100000) in this region^21^, the corresponding power for the dominant OR of rs2057178 was 87.77%. For SNP rs4331426, the minor allele (G allele) frequencies between TB cases and controls showed no statistically significant difference (χ^2^ = 0.04, *P* = 0.8390). The Hardy-Weinberg equilibrium test demonstrated that genotypes of each locus in the controls were all in Hardy-Weinberg equilibrium (χ^2^ = 2.79, *P* = 0.095 for rs2057178 and χ^2^ = 0.142, *P* = 0.704 for rs4331426).

Then, the controls were classified as QFT-positive and QFT-negative to compare the genotype distributions of the two variants among the TB cases, non-infected controls and LTBI controls. The data in Table 2 show that the CT genotype of rs2057178 was significantly associated with a 0.60-fold (adjusted OR = 0.40, 95% CI, 0.22–0.70) decreased risk of TB in QFT-positive controls. The protective effect of the CT genotype was also observed in the QFT-negative controls (adjusted OR = 0.57, 95% CI, 0.36–0.90). The Cochran-Armitage trend test showed that the proportion of the CT genotype was increasing from the QFT-negative controls (8.0%) to the QFT-positive controls (11.4%, *P*_*trend*_  = 0.0006). No associations of rs4331426 with TB were found among the TB cases and the subgroups of the controls.

The liner regression analysis was conducted between the genotypes of the two SNPs and the whole genome mRNA expression levels in the lymphoblastoid cell lines. The 45 CHB were classified into two groups based on the genotypic data of rs2057178 and rs4331426. For rs2057178, 42 CHB had the CC genotype, two CHB had the CT genotype and one CHB failed the genotyping (no TT genotype was found). For rs4331426, 39 CHB had the AA genotype, four CHB had the AG genotype and two CHB failed the genotyping (no GG genotype was found). The GEO2R analyzed the expression levels of 47293 genes in each CHB subject, and 28 genes revealed significantly different expression levels between the two genotype groups of rs2057178 by moderate *t* test after multiple comparison (Table 3). However, the usual *t* test only found the first 20 genes were in relationship with SNP rs2057178. Thus, we included the first 20 genes for both reaching significance. No gene expression levels were found to be associated with the genotypes of rs4331426 after multiple comparison adjustment (data not shown).

## Discussion

In this case-control study, the T allele of SNP rs2057178 was significantly associated with a decreased risk of TB in the Han Chinese population, and 28 mRNA levels were found to be associated with the genotypes of rs2057178 in the 42 CHB, which indicated a potential functional role of rs2057178 in modulating those gene expression levels. However, no genotype of rs4331426 was found to be associated with TB susceptibility and no gene expression levels were associated with any genotype of rs4331426.

Thy *et al.* first reported that rs4331426 was associated with TB susceptibility in a GWAS of the African population in 2010^11^. The HapMap data showed that the G allele frequency of rs4331426 in the Han Chinese population was 0.044, whereas the G allele frequency in the African population was 0.51. Because of the vast difference in the G allele frequencies between the African and Asian populations, the results of repeated association studies would be different. Another replicated association study conducted in the Chinese population by Wang *et al.* found that the G allele of rs4331426 had an opposite effect on TB susceptibility^22^ compared with the results of Thy *et al.* The G allele frequency was 0.0338 in the control group of Wang’s study, while the G allele frequency in our control group was 0.0301, all were close to the G allele frequency of 0.044 of the CHB of the HapMap data. Based on our data, we did not find any association between the genotypes of rs4331426 and TB predisposition. Another two association studies conducted in the Chinese population also did not find a relationship between rs4331426 and TB risk^23^,^24^. As SNP rs4331426 was located in a gene desert region, it was difficult to determine the function of the locus. Generally, it was postulated that other functional loci, in linkage with rs4331426, would be the target loci that were involved in the mechanism of predisposition to TB. In this study, the relationship between the genotypes of rs4331426 and the whole-genome expression levels indicated that no gene expression levels were correlated with the genotypes of rs4331426 after multiple comparison adjustment.

A subsequent GWAS by Thy *et al.* revealed that rs2057178 was associated with TB susceptibility^10^. In Thy’s study, the effect of the T allele on TB susceptibility was further verified in the Gambian and Russian populations. However, the association between rs2057178 and the predisposition to TB failed to replicate in the Indonesian population. Another study conducted in the Asian population also failed to replicate the protective effect of the T allele of rs2057178 on TB susceptibility^23^. It is interesting that a recent GWAS conducted in the African population also revealed the relationship between rs2057178 and TB susceptibility^9^. For the populations discussed above, the HapMap data showed that the T allele frequency of rs2057178 varied with a broad spectrum; it was highest in the African population (0.33) and lowest in the Asian population (0.02). Inter-population heterogeneity cannot be ignored for genetic susceptibility for TB. However, in this study, SNP rs2057178 was found to be significantly associated with TB susceptibility in the Han Chinese population. When the control group was stratified into QFT-positive and QFT-negative groups, the proportion of the CT genotype in the QFT-positive group (11.4%) was higher than that of the QFT-negative group (8%). The Cochran-Armitage trend test revealed that the QFT-positive group with the CT genotype would be more resistant to TB, which suggested that the T allele of rs2057178 might protect people latently infected with TB from developing TB disease. Although the locus was associated with TB susceptibility, the functional role of the locus was not clearly determined as the SNP was located in an intergenic region. SNP rs2057178 was in the 45 Kb downstream of Wilms’ tumor 1 (WT1) gene, which had been shown to be associated with the occurrence of Wilms’ tumor^25^. It was reported that WT1 variants might play a role in altering the effects of interferon-beta on vitamin D^26^, which had been shown to be beneficial in the treatment of TB^27^. Meanwhile, the WT1 gene was involved in the activation of the vitamin D receptor^28^, which was critically important for binding with 1,25-dihydroxyvitamin D3 to modulate the immune system in fighting *M.* tb infection^29^.

**Figure 1:**
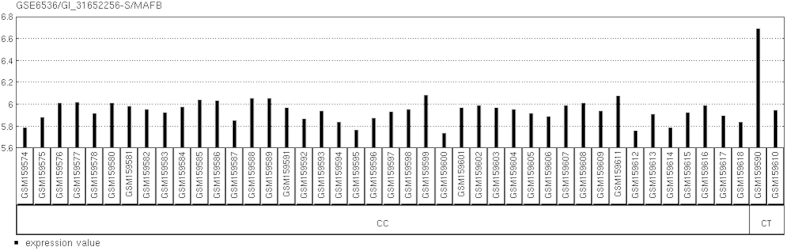
MAFB mRNA expression levels among the different genotypes of rs2057178 of the CHB.

**Figure 2:**
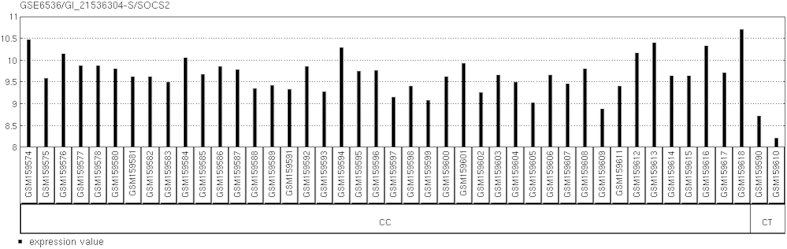
SOCS2 mRNA expression levels among the different genotypes of rs2057178 of the CHB.

Although the HapMap genotypic data of rs2057178 and the whole-genome expression levels of the 42 CHB did not reveal a significant association between the genotypes of rs2057178 and WT1 gene expression, another 20 gene expression levels were found to be significantly associated with rs2057178. More importantly, v-maf avian musculoaponeurotic fibrosarcoma oncogene homolog B (MAFB, Fig. 1), suppressor of cytokine signaling 2 (SOCS2, Fig. 2) were found to be associated with SNP rs2057178 in infectious diseases. According to the fold change (FC) of the gene expressions, MAFB was up expressed by 31% while SOCS2 was down expressed by 57%. MAFB was first reported to be a candidate gene for TB susceptibility in a GWAS by Mahasirimongkol *et al.*^30^, and the expression level of MAFB was found to be higher in patients with active TB compared with the healthy controls and previous TB cases^31^. Our study provided evidence for rs2057178 in modulating the TB susceptible gene MAFB in trans effect, and the mechanism of modulation needs to be further explored.

Simultaneously, the SOCS2 expression level was significantly decreased in the CT genotype of rs2057178 for the CHB compared with the CC genotype. A previous study showed that SOCS2 was required to mediate the effects of lipoxin^32^, which was thought to negatively regulate protective Th1 responses against mycobacterial infection *in vivo*^33^.

**Figure 3:**
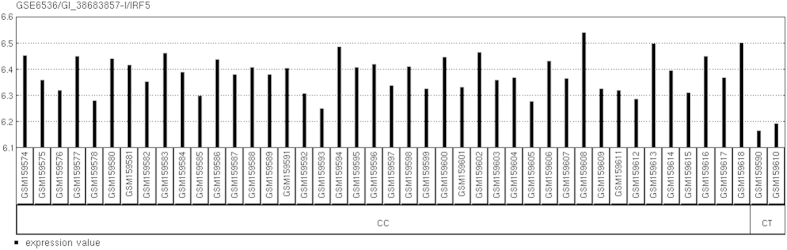
IRF5 mRNA expression levels among the different genotypes of rs2057178 of the CHB.

Even though the interferon regulatory factor 5 (IRF5, Fig. 3) was not found in association with SNP rs2057178 by the usual *t* test, and the FC showed that IRF5 was only slightly decreased (14%) among the CT genotype of rs2057178, IRF5 has an important role in the type 1 interferon response to *M*. tb^34^. Thus, there is a contradiction between the protective effect of the CT genotype of rs2057178 and the decreased expression level of IRF5, and the level of IRF5 needs to be further validated in TB cases and controls.

Several limitations need to be noted in this study. First, the QFT method is an indirect method for detecting latent TB infection, and it may not accurately represent the existence of *M*. tb *in vivo* because we do not know how long the immunological reaction to *M*. tb will last. However, the differentiation ability of the QFT is more convincing in detecting *M*. tb-induced infection rather than other *Mycobacteria* when compared with the tuberculin skin test. Second, the transcriptome varies considerably across different cell populations and developmental stages. A previous study revealed the different cell-type associated gene expression profiles of tuberculosis^35^. Even some researchers found that the interferon-inducible genes were predominantly expressed in neutrophils and, to some extent, in monocytes, but not in T cells^36^. Gene expression levels in other cell types should be evaluated to comprehensively reveal the potentially distinct gene expression profiles of different cell populations. Third, the limited sample size for revealing the associations between SNPs and gene expression levels may be confounded by other factors, and it is worthwhile to compare the actual mRNA expression levels among the TB cases and the healthy controls with larger samples in future studies.

In conclusion, we replicated the loci of TB GWAS in the Han Chinese population. We found that rs2057178 was significantly associated with TB predisposition and that the expression levels of MAFB and SOCS2 were significantly associated with the genotypes of rs2057178. We assume that MAFB and SOCS2 could be potential candidate genes for TB susceptibility in the Han Chinese population. Further functional studies are required to reveal the mechanism of host genetics on TB susceptibility. Additionally, the liner regression analysis for the association between SNP genotypes and gene expression levels could be a choice for exploring the potential functional role of disease predisposition loci.

## Additional Information

**How to cite this article**: Chen, C. *et al.* A rare variant at 11p13 is associated with tuberculosis susceptibility in the Han Chinese population. *Sci. Rep.* **6**, 24016; doi: 10.1038/srep24016 (2016).

## Figures and Tables

**Table 1:** The genotypes distribution of rs2057178 and rs4331426 between tuberculosis cases and controls.

Genotypes	Controls (N = 763) n(%)	Cases (N = 763) n(%)	Crude ORs and 95% CI	Adjusted ORs and 95% CI*
rs2057178				
CC	692 (90.7%)	724 (94.9%)	1.00	1.00
CT	67 (8.8%)	37 (4.8%)	0.53 (0.35–0.80)	0.52 (0.34–0.78)
TT	4 (0.5%)	2 (0.3%)	0.48 (0.09–2.62)	0.56 (0.10–3.10)
Dominant model CT + TT vs. CC	71 (9.4%)	39 (5.1%)	0.53 (0.35–0.79)	0.52 (0.35–0.78)
T allele frequency	0.048	0.027	χ^2^ = 14.07, *P* = 0.0002	
Hardy-Weinberg equilibrium	χ^2^ = 2.79, *P* = 0.095			
rs4331426				
AA	718 (94.1%)	715 (93.7%)	1.00	1.00
AG	44 (5.8%)	48 (6.3%)	1.10 (0.72–1.67)	1.08 (0.71–1.66)
GG	1 (0.1%)	0	NA	NA
Dominant model AG + GG vs. AA	45 (5.9%)	48 (6.3%)	1.07 (0.70–1.63)	1.06 (0.70–1.62)
G allele frequency	0.030	0.031	χ^2^ = 0.04, *P* = 0.8390	
Hardy-Weinberg equilibrium	χ^2^ = 0.142, *P* = 0.704			

^*^Adjusted by age and gender.

**Table 2:** The genotypes distribution of rs2057178 and rs4331426 between tuberculosis cases and IGRA positive and negative controls.

Genotypes	Cases (N = 763) n(%)	QTF Negative Controls (N = 587) n(%)	Crude ORs and 95% CI	Adjusted ORs and 95% CI*	QTF Positive Controls (N = 176) n(%)	Crude ORs and 95% CI	Adjusted ORs and 95% CI*	*P* trend
rs2057178								
CC	724 (94.9%)	537 (91.5%)	1.00	1.00	155 (88.1%)	1.00	1.00	
CT	37 (4.8%)	47 (8.0%)	0.58 (0.37–0.91)	0.57 (0.36–0.90)	20 (11.4%)	0.40 (0.22–0.70)	0.40 (0.23–0.72)	0.0006
TT	2 (0.3%)	3 (0.5%)	0.49 (0.08–2.97)	0.62 (0.10–3.74)	1 (0.6%)	0.43 (0.04–4.75)	0.30 (0.03–3.46)	
Dominant model CT + TT vs. CC	39 (5.1%)	50 (8.5%)	0.58 (0.38–0.89)	0.57 (0.37–0.89)	21 (12.0%)	0.40 (0.23–0.70)	0.40 (0.23–0.70)	
rs4331426								
AA	715 (93.7%)	547 (93.2%)	1.00	1.00	171 (97.2%)	1.00	1.00	
AG	48 (6.3%)	39 (6.6%)	0.94 (0.61–1.46)	0.93 (0.60–1.44)	5 (2.8%)	2.30 (0.90–5.85)	2.33 (0.91–5.96)	0.2327
GG	0	1 (0.2%)	NA	NA	0	NA	NA	
Dominant model AG + GG vs. AA	48 (6.3%)	40 (6.8%)	0.92 (0.60–1.42)	0.90 (0.58–1.40)	5 (2.8%)	2.30 (0.90–5.85)	2.33 (0.91–5.96)	

^*^Adjusted by age and gender.

**Table 3:** The genotypes of rs2057178 in association with specific gene expressions.

ID	Adjusted P for moderate *t* test*	Adjusted P for usual *t* test*	Fold Change	Gene.title	Gene.symbol
GI_31377766-S	0.058	0.087571289	0.5	polymerase (RNA) II (DNA directed) polypeptide M	POLR2M
GI_33667071-S	0.063	0.09215385	0.50698	CD300a molecule	CD300A
GI_12545379-S	0.063	0.09215385	1.580083	secretin	SCT
GI_7705930-S	0.09	0.098064729	0.378929	HECT and RLD domain containing E3 ubiquitin protein ligase 5	HERC5
GI_27312030-S	0.09	0.098064729	1.164734	tumor suppressor candidate 5	TUSC5
GI_27545446-S	0.09	0.112547153	0.547147	vesicle-associated membrane protein 7	VAMP7
GI_29789067-S	0.127	0.156789692	1.42405	family with sequence similarity 19 (chemokine (C-C motif)-like), member A5	FAM19A5
GI_24497598-A	0.131	0.162399477	0.673617	phosphatidylinositol glycan anchor biosynthesis, class P	PIGP
GI_42542393-S	0.131	0.165706023	0.68302	lysophosphatidylcholine acyltransferase 3	LPCAT3
GI_18491001-S	0.131	0.162399477	0.888843	WNT1 inducible signaling pathway protein 2	WISP2
GI_4885286-S	0.131	0.165706023	0.702222	guanine nucleotide binding protein (G protein), gamma 5	GNG5
GI_24307950-S	0.131	0.165706023	0.582367	tudor domain containing 7	TDRD7
GI_4758669-S	0.131	0.165706023	0.594604	leupaxin	LPXN
GI_31652256-S	0.131	0.165706023	1.310393	v-maf avian musculoaponeurotic fibrosarcoma oncogene homolog B	MAFB
GI_19924155-A	0.14	0.173671278	0.752623	regulatory factor X-associated ankyrin-containing protein	RFXANK
GI_27734778-S	0.149	0.191442937	0.823591	DTW domain containing 2	DTWD2
GI_21536304-S	0.149	0.194057035	0.426317	suppressor of cytokine signaling 2	SOCS2
GI_40353206-S	0.149	0.194057035	0.554785	MRG/MORF4L binding protein	MRGBP
GI_6912277-S	0.149	0.194057035	0.641713	COMM domain containing 3	COMMD3
GI_4757769-S	0.149	0.194057035	0.607097	ras homolog family member H	RHOH
GI_7019372-S	0.149	0.200727205	0.528509	fasciculation and elongation protein zeta 2 (zygin II)	FEZ2
GI_22212923-I	0.177	0.220792973	0.773782	nuclear transcription factor, X-box binding 1	NFX1
GI_8922698-S	0.177	0.220792973	0.574349	CNDP dipeptidase 2 (metallopeptidase M20 family)	CNDP2
GI_37059763-S	0.177	0.220792973	0.550953	gephyrin	GPHN
GI_38683857-I	0.185	0.220792973	0.864537	interferon regulatory factor 5	IRF5
GI_34222319-S	0.185	0.235007739	0.524858	Josephin domain containing 1	JOSD1
GI_42794768-S	0.198	0.235007739	0.550953	p21 protein (Cdc42/Rac)-activated kinase 1	PAK1
GI_23397652-S	0.199	0.235007739	0.668964	phosphatidylinositol glycan anchor biosynthesis, class T	PIGT

^*^Adjusted by Benjamini & Hochberg.
